# Characterizing the clinical implementation of a novel activation-repolarization metric to identify targets for catheter ablation of ventricular tachycardias using computational models

**DOI:** 10.1016/j.compbiomed.2019.03.018

**Published:** 2019-05

**Authors:** Fernando O. Campos, Michele Orini, Peter Taggart, Ben Hanson, Pier D. Lambiase, Bradley Porter, Christopher Aldo Rinaldi, Jaswinder Gill, Martin J. Bishop

**Affiliations:** a School of Biomedical Engineering and Imaging Sciences, King's College London, London, United Kingdom; b The Heart Hospital, University College London, London, United Kingdom; c Institute of Cardiovascular Science, University College London, London, United Kingdom; d Department of Mechanical Engineering, University College London, London, United Kingdom; e Electrophysiology Department, Barts Heart Centre, St Bartholomew's Hospital, London, United Kingdom; f Department of Cardiology, Guys and St Thomas' NHS Trust, London, United Kingdom

**Keywords:** Arrhythmia, Ventricular tachycardia, Ablation, Computer simulation

## Abstract

Identification of targets for catheter ablation of ventricular tachycardias (VTs) remains a significant challenge. VTs are often driven by re-entrant circuits resulting from a complex interaction between the front (activation) and tail (repolarization) of the electrical wavefront. Most mapping techniques do not take into account the tissue repolarization which may hinder the detection of ablation targets. The re-entry vulnerability index (RVI), a recently proposed mapping procedure, incorporates both activation and repolarization times to uncover VT circuits. The method showed potential in a series of experiments, but it still requires further development to enable its incorporation into a clinical protocol. Here, *in-silico* experiments were conducted to thoroughly assess RVI maps constructed under clinically-relevant mapping conditions. Within idealized as well as anatomically realistic infarct models, we show that parameters of the algorithm such as the search radius can significantly alter the specificity and sensitivity of the RVI maps. When constructed on sparse grids obtained following various placements of clinical recording catheters, RVI maps can identify vulnerable regions as long as two electrodes were placed on both sides of the line of block. Moreover, maps computed during pacing without inducing VT can reveal areas of abnormal repolarization and slow conduction but not directly vulnerability. In conclusion, the RVI algorithm can detect re-entrant circuits during VT from low resolution mapping grids resembling the clinical setting. Furthermore, RVI maps may provide information about the underlying tissue electrophysiology to guide catheter ablation without the need of inducing potentially harmful VT during the clinical procedure. Finally, the ability of the RVI maps to identify vulnerable regions with specificity in high resolution computer models could potentially improve the prediction of optimal ablation targets of simulation-based strategies.

## Highlights

• Safe and accurate detection of targets for catheter ablation remains a challenge.
• We conducted a thorough assessment of the Re-entry Vulnerability Index (RVI).
• Parameters of the algorithm can alter the specificity and sensitivity of RVI maps.
• When constructed on sparse grids RVI maps could still detect arrhythmogenic sites.
• In absence of arrhythmia, RVI maps revealed abnormal sites, but not vulnerability.

## 1 Introduction

Ventricular tachycardias (VTs) carry the greatest risk of sudden death in patients with ischemic heart disease [1]. VTs are often driven by re-entrant electrical wavefronts which are sustained by isthmuses formed by diseased surviving myocyte bundles within the scar [2]. Radiofrequency catheter ablation remains the only potential curative treatment for ischemic scar-related VTs [3]. However, success rates are highly depended on the ability to accurately locate the VT circuit [4].

Identification of the re-entrant entry/exit points often requires VT-induction to delineate isthmuses from activation mapping. However, inducibility may be neither possible nor desired as it increases the risk of the procedure. In this case, voltage-mapping is performed to uncover abnormal substrates within the scar [3], regions of local abnormal ventricular activity [[5], [6], [7]], discrete slow conducting channels [[8], [9], [10], [11]], or areas with abnormal signal amplitude during either sinus or paced rhythm [4]. Algorithms for identifying a VT exit based on the 12-lead ECG characteristics have also been assessed [[12], [13], [14]]. However, the area of heart muscle targeted for ablation by substrate mapping often results in larger lesions that can further impair ventricular function. Nevertheless, repolarization, a key factor in the formation of a re-entrant circuit, is often neglected during mapping techniques hindering the identification of critical sites and increasing the risk of later recurrence of VT.

Our group has recently developed a novel substrate mapping procedure, termed the Re-entry Vulnerability Index (RVI), which incorporates both activation (AT) and repolarization (RT) times to identify regions of high susceptibility to re-entry [15] (see Section 2.1.1 for details). The RVI metric quantitatively assesses the likelihood of re-entrant wavefront-waveback interactions by computing the time interval between the arrival of the wave at the exit site and the regaining of excitability (repolarization) of tissue just proximal to it [16,17]. The algorithm has been shown to reliably identify the region of re-entry in a clinical case of scar-related VT [15] as well as the earliest endocardial activation site of VT in patients with right ventricular pathology [18]. Furthermore, *in-silico* investigations with realistic infarct scar anatomy models demonstrated that simulated ablation of vulnerable regions detected by the RVI successfully prevented the re-entry re-initiation [19]. Despite such promising results, many questions regarding the RVI mapping algorithm remain to be addressed to enable its incorporation into a clinical protocol at practical time scales. First, although the definition of the RVI metric between a pair of recording electrodes is well defined, different forms of mapping RVI values globally have been employed [15,18,19]. These may significantly change the quantitative features of an “RVI Map”. Second, the density of recording sites are highly dependent on the mapping system and might affect the ability of the RVI metric to highlight regions of re-entry susceptibility. Third, the RVI has been shown to accurately identify regions of conduction block and re-entry following premature stimulation [15,19]. However, re-entry induction may not be achieved or safe for the patient. Moreover, the use of premature pacing protocols increases the duration of the mapping procedure. Finally, the effect of electrophysiological changes occurring in the infarct border zone (BZ) at different stages of ischemic heart disease on RVI maps has not been evaluated.

The goal of this study is to use computational models to mechanistically address all aforementioned questions in order to optimize the RVI algorithm for its use within a clinical ablation procedure. Idealized as well as anatomically-accurate infarct models are used here to gain an in-depth understanding of how key parameters of the global RVI mapping, such as interpolation between a recording site and its downstream neighbors and search radius, may be improved. RVI maps are computed on sparse recording grids resembling clinically-relevant mapping conditions. Different pacing protocols are employed to assess the ability of RVI maps to identify vulnerable regions in absence of block/re-entry. Variations in electrophysiological properties of the BZ are also tested as they are more easily altered in *in-silico* experiments than in experimental or clinical investigations.

## 2 Methods

### 2.1 Global RVI map construction

#### 2.1.1 Principles of the RVI calculation

**Fig. 1:**
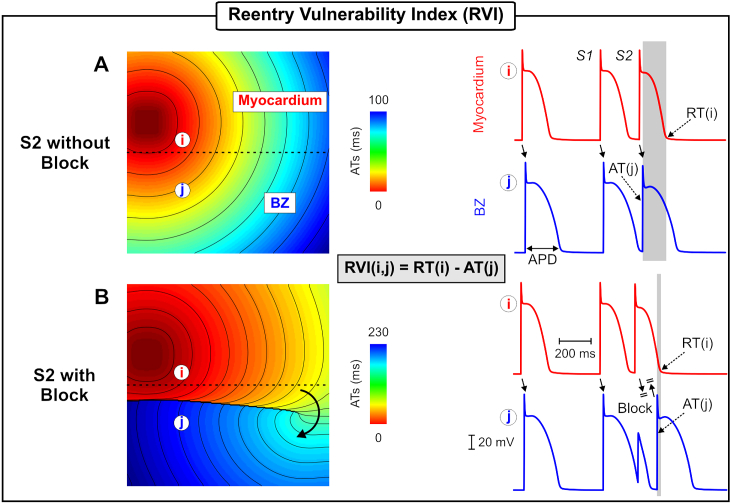
Illustration of the RVI metric in normal conduction as well as in block. A) Activation sequence following an S2 beat that propagates throughout the tissue (S2 without block). Right panel: APs from the myocardium (red) and BZ (blue). The RVI at recording site *i* is given as the time difference between the RT(i) of the premature S2 beat and time of arrival of the S2 wavefront at the distal site AT(j). B) Activation sequence following an S2 beat with a shorter coupling interval leading to conduction block. The S2 beat blocks in the BZ where tissue is still refractory. The wavefront travels around the line of block and back towards the proximal site. Changes in the RVI at measurement site *i* value is illustrated by the thickness of the vertical gray bars which is large in normal propagation (A) and small in the case of block (B).

The RVI metric initially proposed by Coronel et al. [16,17] is a quantitative measurement of the likelihood of wavefront-waveback interactions around a re-entrant circuit. A re-entrant circuit is formed when an electrical wavefront encounters a region, such as the infarct border zone (BZ), that has not yet repolarized leading to conduction block. However, the wavefront can still travel along the line of block and find tissue that has regained excitability, enabling it to re-enter the tissue of origin from the distal side of the line of block. If the proximal tissue, from where the wavefront originated, has repolarized then a re-entrant circuit ensues. Thus, the time interval between the repolarization of the proximal region and the activation of the distal side of the line of block determines whether re-entry occurs [16] and is the basis for the RVI algorithm. Fig. 1 illustrates how the RVI is calculated following a premature S2 stimulus. Repolarization heterogeneity in this example is due to differences in action potential (AP) duration (APD) between the myocardium (shorter APD - red traces) and BZ (longer APD - blue traces) [20]. The RVI between the pair of recording electrodes *i* and *j* is defined to be the difference between the RT of *i* and the AT of *j*:

(1) RVI(i,j)=RT(i)−AT(j)

In Fig. 1A, the premature S2 wavefront successfully travels from the myocardium to the BZ as the latter had recovered excitability. In this scenario, RVI(i,j) comes close to the APD of the myocardium as highlighted in gray on the right panel of Fig. 1A. Fig. 1B illustrates a scenario where a premature S2 beat following a shorter coupling interval initiated in the myocardium fails to propagate into the BZ, but travels along the line of block until it encounters tissue that has recovered excitability. The S2 wave travels back from the distal side towards the myocardium where it originated. In this case, magnitude and sign of RVI(i,j) will depend on whether the attempt of the wavefront to re-enter at the starting point is successful or not. If the tissue under recording site *i* has not completely repolarized, RVI(i,j) will be small but positive (bidirectional block; Fig. 1B). However, if the tissue at *i* repolarizes earlier, or if the S2 beat travels slower in the BZ (increasing AT at *j*), it will re-enter the myocardium and RVI(i,j) will be negative. Therefore, the RVI can measure the tissue susceptibility to re-entry and although counterintuitive, lower RVI values are associated with a higher susceptibility.

#### 2.1.2 RVI on a grid of measurement points

Child et al. [15] proposed an algorithm to map the RVI metric globally in order to identify spatial regions with high susceptibility to re-entry. First, ATs and RTs of the S2 beat are derived for all recording sites. Second, for a given recording site *i* all other sites *j* that are activated later than site *i* (*i.e.* are downstream neighbors of *i* and lie within a prescribed search radius (R) are found. Third, the RVI for each recording site pair RVI(i,j) as in Eq. (1) is calculated. Fourth, all RVIs associated with *i* are interpolated using one of the three following methods:

1 Nearest Neighbor: following our previous computational study [19], the RVI(i,j) value is associated with the nearest neighbor of the geometric midpoint *k* between the pair of points *i* and *j*. If *k* is the same midpoint between different pair of electrodes, then the final RVI is given as the mean of all values associated with *k*;
2 Average: the mean of all RVIs(i,j) is computed and associated with node *i*;
3 Minimum: the minimum RVI among all RVI(i,j) is found and associated with node *i*. This approach has been recently used to assess arrhythmogenesis in right ventricular disorders [18].

Finally, a color map is constructed to highlight small or negative RVI values to reveal the regions most vulnerable to re-entry. Here, the effect of these three different interpolation methods on the spatial RVI map will be investigated.

### 2.2 Computational models

Two geometrical finite element (FE) models were used in this study to simulate electrical activity in cardiac tissue: a 2D sheet with an idealized representation of infarct scars and BZ and a rabbit biventricular (BiV) model including an anatomically-detailed infarct.

#### 2.2.1 2D idealized model

**Fig. 2:**
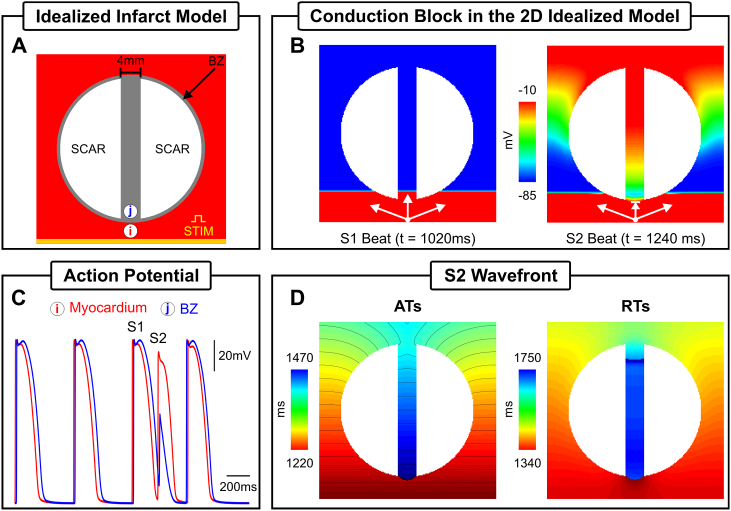
Simulation results within the 2D computational model. A) Idealized infarct model consisting of: healthy myocardium (red); a circular region representing the BZ (gray); and two circular segments representing the necrotic scar (white). The BZ region contains an isthmus of 4 mm width separating the scars. B) Spatial distribution of $V_{m}$ at the times the last S1 (t = 1020 ms) and S2 (t = 1240 ms) beats arrive at the isthmus' proximal mouth. C) Time course of $V_{m}$ at recording sites (*i*) and (*j*) located at the myocardium and BZ inside the isthmus, respectively. D) ATs and RTs of the premature S2 beat.

An idealized infarct was generated in a 4 × 4 cm sheet discretized at 200 *μ*m resolution (40,000 quadrilateral FEs). The infarct region was comprised of two circular segments representing the scar separated by a 4 mm conducting isthmus. The schematic of the 2D idealized infarct models is illustrated in Fig. 2A. The radii of the scar and BZ were set to 15 and 15.5 mm, respectively, giving a transition distance of 500 *μ*m between the scar and healthy myocardium. Ionic membrane dynamics were simulated with Mahajan-Shiferaw (MSH) rabbit ventricular cell model [21]. The tissue within the BZ had its ionic properties adjusted to produce a lengthened APD [20]. Specifically, the conductance of the rapid delayed rectifier potassium current (gKr) was reduced to 30% and the conductance of the slow delayed rectifier potassium current (gKs) was reduced to 20%. If not stated otherwise, the whole tissue (myocardium and BZ) bulk conductivity tensor $\sigma_{m}$ (see Eq. (2)) in the model was set to be isotropic with a value of 0.068 S/m, ensuring a re-entrant circuit could fit within the 2D sheet. The scar tissue was represented as being necrotic, *i.e.*, by imposing no-flux boundary condition at its interface.

#### 2.2.2 BiV scar anatomy model

**Fig. 3:**
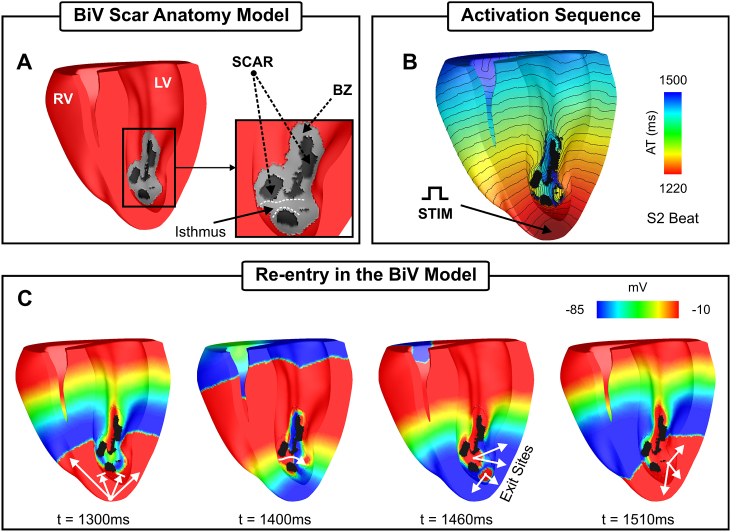
Simulation results within the BiV computational model. A) BiV model with a clipping plane view highlighting the endocardial surface of both right (RV) and left (LV) ventricles. Inset: intramural infarct anatomy with the delineation of one of the isthmuses within the scar. B) Activation sequence of the S2 beat following apical stimulation. C) Spatial distribution of $V_{m}$ at different times illustrating conduction block of the S2 wavefront (t = 1300 ms); propagation through the isthmus highlighted in A) at t = 1400 ms; and the exit sites of the re-entry (t = 1460 ms).

The rabbit BiV model employed in this study is comprised of 547,680 myocardial nodes defining 3,073,529 tetrahedral FEs with a mean discretization of 279 *μ*m and contained realistic fiber architecture derived from histological information [22]. The BiV model includes an anatomically-accurate infarct scar and BZ representing approximate infarct regions created following occlusion of the left anterior descending coronary artery [19] (see Fig. 3A). The intracellular domain, comprising of both myocardium and BZ, was modelled with anisotropic bulk conductivities values of 0.068 and 0.009 S/m along and transverse to the fiber direction, respectively. Within BZ regions, $\sigma_{m}$ was set to be isotropic and reduced by 90% to replicate known conduction delays through these pathological regions [23]. As in the 2D idealized model, the MSH model was used to represent membrane dynamics with the same modifications to ionic currents to produce a prolonged APD in the BZ to facilitate the unidirectional block.

### 2.3 Governing equations

Cardiac electrical activity within the geometrical models was simulated using the monodomain formulation:

(2) $$\nabla \cdot \left(\sigma_{m}\nabla V_{m}\right)=\beta I_{m},$$

(3) $$C_{m}\frac{\partial V_{m}}{\partial t}+I_{ion}\left(V_{m},\eta \right)-I_{stim}=I_{m},$$

(4) $$\frac{\partial \eta }{\partial t}=f\left(V_{m},\eta \right)$$

where $\sigma_{m}$  = diag($\sigma_{ml}$, $\sigma_{mt}$, $\sigma_{mt}$) is the harmonic mean conductivity tensor or the effective bulk conductivity [24]; $V_{m}$ is the transmembrane voltage; *β* = 0.14 *μ*m^−1^ is the surface to volume ratio; $I_{m}$ is the transmembrane current density; $C_{m}$ is the membrane capacitance per unit area; $I_{ion}$ is the density of the total ionic current flowing through the membrane channels, pumps and exchangers [21]; and $I_{stim}$ is the stimulus current density. $I_{ion}$ depends on $V_{m}$ as well as on *η*, a vector of state variables describing channel gating and ionic concentrations. Tissue electrical dynamics was simulated within the geometrical models using the Cardiac Arrhythmia Research Package (CARP) [25] (http://carp.meduni-graz.at).

### 2.4 Pacing protocol

Electrical activity was initiated in both geometrical models following the S1-S2 protocol illustrated in Fig. 1. Unless otherwise specified, three S1 beats at a basic cycle length (BCL) of 500 ms followed by a premature S2 with a coupling interval of 220 ms were simulated. In the 2D idealized infarct model, the S1-S2 pacing was applied in the lowermost portion of the tissue (Fig. 2A) while the BiV model was paced at the apex (Fig. 3B).

### 2.5 Data analysis

**Fig. 4:**
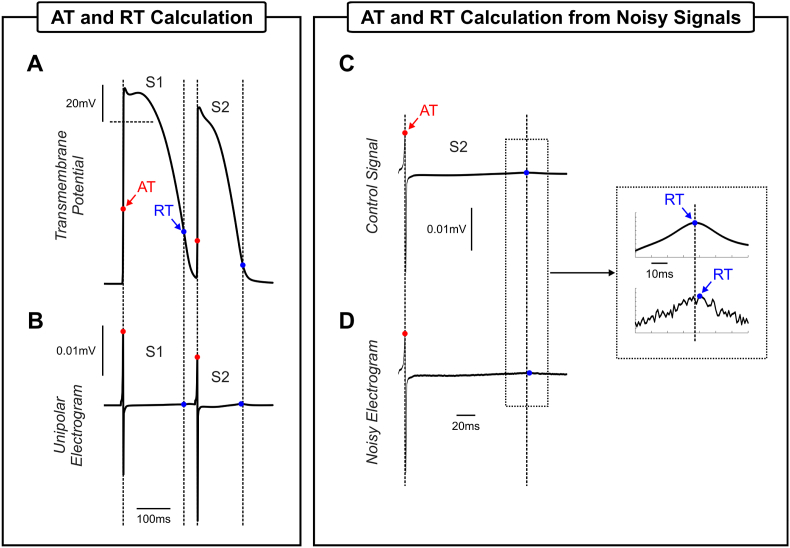
AT and RT calculation. ATs (red dots) and RTs (blue dots) obtained from: A) APs and B) electrograms computed following the last S1 beat and the S2 at recording site *i* in Fig. 2A. Electrogram of the premature S2 beat C) without noise and D) with noise. Dash lines represent projections of the AP time markers on the electrogram.

ATs and RTs were derived for all recording sites as the times at which $V_{m}$ crossed −20 mV (with positive gradient) and −70 mV with (negative gradient), respectively. As illustrated in Fig. 4A–B, AT and RT closely reflect common surrogates markers obtained from simulated unipolar electrograms [26], that are typically recorded by electrophysiology catheters. Recording grids with different densities were assessed:

• High-density grid: each finite element node in the respective model was considered as a recording electrode;
• Decapolar catheter: 10 aligned recording electrodes with 2-8-2 mm spacing if not stated otherwise;
• Multipolar catheter: to reproduce data collection in the lab using a multipolar catheter, 50 random points located on the surface of the respective tissue model were randomly selected as the center of a cluster of recording sites. Each cluster consisted of 5% of all nodes (also randomly selected) located within a sphere of 1.5 mm radius.

Unlike in the simulated traces shown in Fig. 4A–B, signals recorded with electrophysiology catheters are often contaminated by noise (see Fig. 4C–D). In order to investigate how the RVI is affected by noise, Gaussian noise [27] was added to AT and RT maps.

## 3 Results

### 3.1 Scar-related re-entry simulation

The S1-S2 protocol illustrated in Fig. 1 (lower panel) was used to induce VT in the *in-silico* infarct models in Figs. 2A and 3A. The vulnerable regions, *i.e.*, sites where block and subsequent re-entry occurred are considered the “ground truth” in this study. Wavefront-waveback interactions next to these sites, the basis of the RVI metric, are presented below. Activation and repolarization sequences resulting from the premature S2 wavefront were used in the following Sections to construct RVI maps and assess their ability to locate scar-related re-entrant circuits.

### 3.2 2D idealized model

Fig. 2B shows spatial distribution of $V_{m}$ at the time the S1 and S2 beats arrive at the proximal mouth of the isthmus, respectively. The S1 wavefront propagates through the isthmus whereas the S2 blocks at the proximal mouth. Fig. 2C illustrates the time course of $V_{m}$ during successful propagation and block at recording points *i* and *j*, located in the healthy myocardium (red trace) and BZ (blue trace), respectively. Conduction block occurs because of the lengthened APD assigned to cells in the BZ, meaning that tissue in this region is still refractory following the S1 stimulus upon arrival of the S2 wavefront (see Fig. 2B). Fig. 2D shows ATs and RTs of the S2 beat in the idealized model. It can be seen from the AT sequence that the S2 wavefront propagates around the non-conducting scar entering at the distal mouth where tissue has regained excitability. Next, it propagates downwards through the isthmus towards the proximal mouth and exits to the myocardium setting up a re-entrant circuit that lasts for one cycle (see Supplemental Video 1 for further details). The proximal mouth of the isthmus (lined arrow in Fig. 2B) is the vulnerable region to VT in the idealized 2D model.

Supplementary video related to this article can be found at https://doi.org/10.1016/j.compbiomed.2019.03.018.

The following is the supplementary data related to this article:

Supplemental Video 1 1 Supplemental Video 1

#### 3.2.1 BiV scar anatomy model

Fig. 3B shows the activation sequence following a premature S2 beat simulated within the BiV model. Like the idealized model in Fig. 2, the S2 beat initially blocks at the BZ because of the prolonged APD defined within cells in this region (see Fig. 2C). Conduction block and re-entry of the S2 wavefront are summarized in Fig. 3C, but can be better appreciated in Supplemental Video 2. At time t = 1300 ms the wavefront blocks at the BZ proximal to the stimulus site, but propagates around the infarct towards the base. At time t = 1400 ms the tissue inside the isthmus depicted in Fig. 3A (inset) regained excitability allowing the wavefront to enter the infarct region from where it propagates slower towards the left ventricle (LV). Around t = 1460 ms the healthy myocardium recovers excitability allowing the S2 beat to exit to the LV (t = 1510 ms). A second exit site resulting from a transmural component of the wavefront can also be seen. See Supplemental Video 2 for additional details of the S1, S2 and the non-sustained re-entrant wavefronts in the BiV model. Unlike in the idealized model, the precise localization of the regions vulnerable to block and re-entry in the BiV are more complex to identify due to the 3D/transmural anatomy of the infarct. Albeit the wavefront blocks at the two lined arrows in Fig. 3C (t = 1300 ms), it only re-enters at the region of block in the LV. Therefore, only the regions labeled exit sites (t = 1460 ms) are vulnerable to VT.

Supplementary video related to this article can be found at https://doi.org/10.1016/j.compbiomed.2019.03.018

The following is the supplementary data related to this article:

Supplemental Video 2 Supplemental Video 2

### 3.3 RVI map construction and its effect on vulnerable region identification

In this Section, the RVI metric is applied to the *in-silico* experiments presented above to identify sites vulnerable to scar-related re-entry. To account for differences in clinical data collection, implementation and interpretation of the RVI algorithm, spatial RVI maps were constructed using different forms to interpolate single RVI values between a central recording site and its downstream neighbors. Specifically, three different interpolation methods are assessed here, as described in the Methods (see Sect. 2.1.2).

**Fig. 5:**
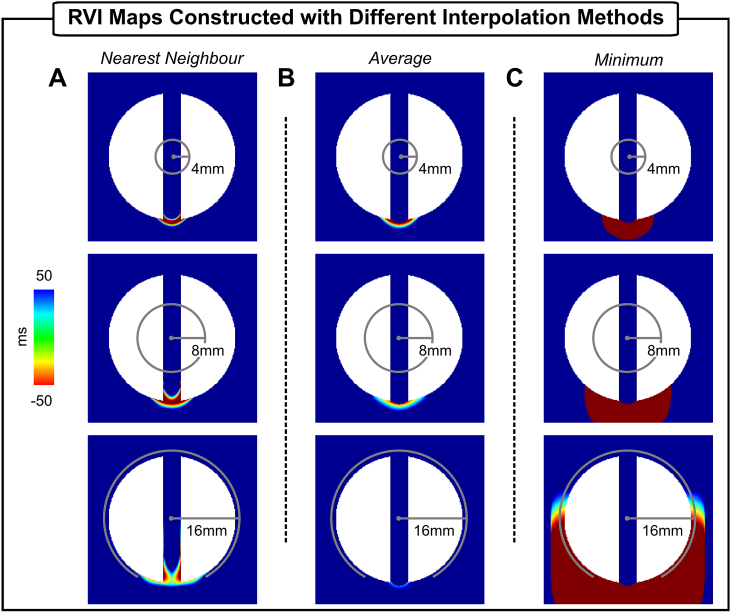
The RVI metric applied to the 2D idealized infarct model. RVI maps constructed using three different interpolation methods: A) nearest neighbor; B) average; and C) minimum. Maps were constructed with search radius R of 4 mm (top panels), 8 mm (mid panels) and 16 mm (bottom panels).

Fig. 5 shows RVI maps of the S2 beat constructed using the nearest neighbor, average and minimum interpolation approaches, respectively, with different search radius R (4 mm, 8 mm and 16 mm). Quantitative information about the vulnerable region (size and lowest RVI value) detected by each method is summarized in Table 1, Table 2. Note that all three maps with R = 4 mm (topmost row) highlight the region of low RVI, which distinctly coincides with the site of conduction block and re-entry at the proximal mouth of the isthmus, described above (Fig. 2D). However, the minimum interpolation (Fig. 5C top) results in a larger “hot-spot” area with more negative RVI values (49.9 mm^2^ and lowest value of −128 ms) than the nearest neighbor (13.8 mm^2^/−99 ms) and the average methods (16 mm_2_/−96 ms). The size of R determines the number and distance of “nearest” downstream neighbors used in the computation of the RVI metric at the recording site in question, which can have important implications on the RVI map (see Methods for details). The dependency of the RVI spatial distribution on R can also be seen in Fig. 5 from top to bottom.

**Table 1:** Size of the vulnerable region (RVI < 50 ms) in the 2D idealized infarct model.

R	Interpolation		
	*Nearest Neighbor*	*Average*	*Minimum*
4 mm	13.8 mm^2^	16.0 mm^2^	49.9 mm^2^
8 mm	28.0 mm^2^	26.4 mm^2^	122.6 mm^2^
16 mm	48.7 mm^2^	8.2 mm^2^	429.0 mm^2^

**Table 2:** Lowest RVI value in the 2D idealized infarct model.

R	Interpolation		
	*Nearest Neighbor*	*Average*	*Minimum*
4 mm	−99 ms	−96 ms	−128 ms
8 mm	−81 ms	−58 ms	−128 ms
16 mm	−60 ms	15 ms	−128 ms

The spatial region with low RVI values (< 50 ms, see Table 1, Table 2) computed using the nearest neighbor interpolation (Fig. 5A) extends from 13.8 mm_2_ to 28 mm_2_ as R increases from 4 mm to 8 mm, but becomes more “blurred” when R is increased to 16 mm as the number of negative RVIs decreases. This is due to averaging effects of measurement points detected as nearest neighbors of multiple pairs of electrodes in the high-density grid, which would not occur using clinical spatial sampling density. This is also reflected in the overall lowest RVI value which becomes less negative: -99 ms (R = 4 mm) and −60 ms (R = 16 mm). Such effect is more pronounced when the average interpolation is used (see Fig. 5B and Table 1, Table 2). Note that the size of the region with small RVI values shrinks as R increases becoming barely visible in the case where R = 16 mm. Moreover, the lowest RVI becomes more positive with a larger R because of averaging small and larger RVIs to a single point. Unlike the nearest neighbor and the average interpolation, in the minimum interpolation the area with negative RVI values monotonically becomes larger with R as shown in Fig. 5C (from top to bottom). As can be seen Table 1, the size of the vulnerable region identified by the minimum extended by factor of 8.6 when R is increased from 4 mm to 16 mm. This is because for the same RT at a recording point, further away sites with late ATs were found to be within R reducing thus the RVI value at that point. However, as shown in Table 2 the lowest RVI value does not change as R increases. This is because the lowest possible RVI value (shortest RT near the line of block minus the latest AT) is found next to the proximal mouth as shown in Fig. 2D.

**Fig. 6:**
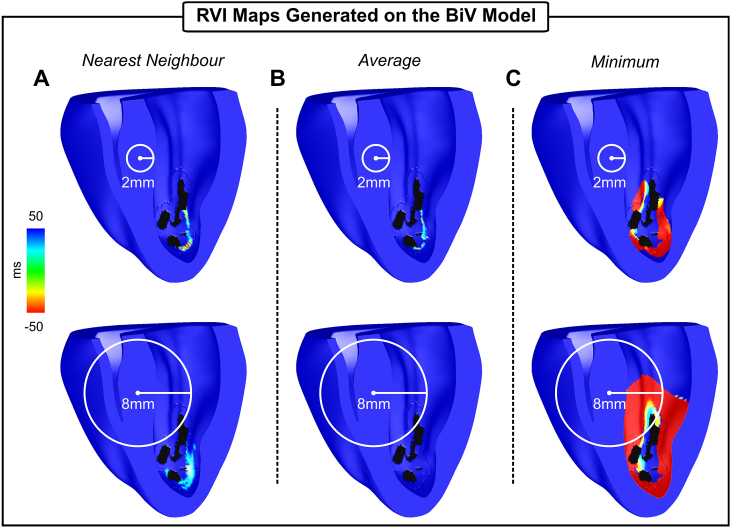
The RVI metric applied to the BiV scar anatomy model. RVI maps constructed using three different interpolation methods: A) nearest neighbor; B) average; and C) minimum. Maps were constructed with R = 2 mm (top panels) and R = 8 mm (bottom panels).

The ability of the RVI metric to detect vulnerable regions in the context of a more complex scar anatomy is now examined in the BiV model. Fig. 6 and Table 3, Table 4 summarize the results obtained employing the three interpolation methods with R of 2 mm and 8 mm. Like the 2D model, the nearest neighbor and the average methods highlight the vulnerable regions with more specificity (see sizes of the identified regions in Table 3) than the minimum interpolation (top panel). Increasing the radius from 2 mm to 8 mm had a more pronounced effect on the map constructed using the average as negative RVI values (minimum of −27 ms for R = 2 mm) were averaged out (minimum of 34 ms for R = 8 mm) during the interpolation. As can be seen in Table 3, the minimum is more sensitive to the increase in R than the other two methods. The volume of tissue detected by the minimum interpolation increased from 13.4 mm^3^ to 89.2 mm^3^, *i.e.* by 6.7, when R increased from 2 mm to 8 mm. Note that in the minimum interpolation with the largest R (Fig. 6C, bottom panel) the entire BZ as well as a part of the healthy myocardium have negative RVIs. The minimum RVI value also become more negative: from −104 ms to −208 ms for R of 2 mm and 8 mm, respectively (see Table 4). Overall, the results obtained with the anatomically-detailed BiV infarct model are consistent with the idealized geometry in Fig. 5.

**Table 3:** Size of the vulnerable region (RVI < 50 ms) in the BiV scar anatomy model.

R	Interpolation		
	*Nearest Neighbor*	*Average*	*Minimum*
2 mm	1.8 mm^3^	1.3 mm^3^	13.4 mm^3^
8 mm	5.0 mm^3^	0.1 mm^3^	89.2 mm^3^

**Table 4:** Lowest RVI value in the BiV scar anatomy model.

R	Interpolation		
	*Nearest Neighbor*	*Average*	*Minimum*
2 mm	−41 ms	−27 ms	−104 ms
8 mm	−6 ms	34 ms	−208 ms

### 3.4 Effect of noise on RVI maps

**Fig. 7:**
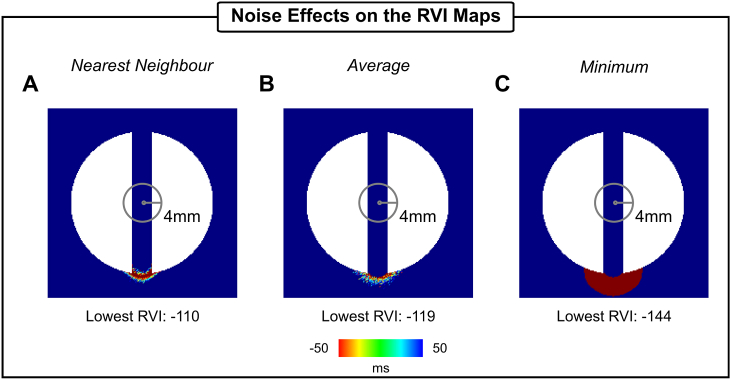
Effects of Gaussian noise on RVI maps. RVI maps constructed with R = 4 mm and three different interpolation methods: A) nearest neighbor; B) average; and C) minimum. Lowest RVI value of each map is also shown.

To gauge the robustness of the RVI maps in Fig. 5, noise with similar characteristics as observed under experimental/clinical conditions was added to the AT and RT maps. Fig. 7 shows the effects of Gaussian noise on RVI maps computed using the nearest neighbor, average and minimum interpolations with R = 4 mm. Compared to Fig. 5 (top panels), it can be seen that the noise blurred the RVI maps constructed with the nearest neighbor and average interpolations, but not with the minimum. However, the lowermost RVI value became more negative for all three cases.

### 3.5 Effect of catheter measurement location on RVI maps

**Fig. 8:**
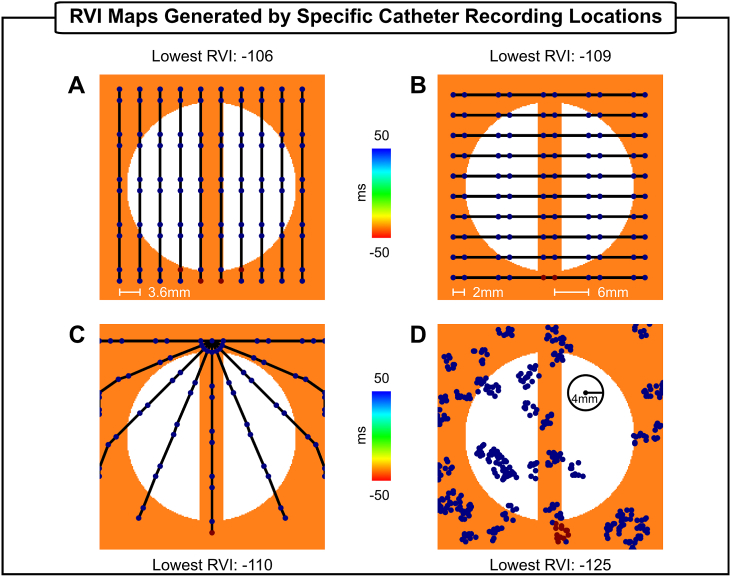
RVI maps computed on different electrode arrangements. A) Decapolar catheters placed parallel to the isthmus. B) Decapolar catheters placed perpendicular to the isthmus. C) Decapolar catheters arranged in a fan-like structure. D) Multipolar catheters placed randomly on the tissue surface. The minimum interpolation and R = 4 mm were used to compute RVIs.

As the RVI metric is intended for use within a clinical ablation procedure, RVI maps are computed on sparse grids resembling various positions of a recording catheter to mimic clinically-relevant mapping conditions. Fig. 8 shows RVI maps obtained with different catheter arrangements using the minimum interpolation. In Fig. 8A–B, RVIs were computed on decapolar catheters (electrode spacing: 2-8-2 mm) aligned parallel, as well as perpendicular, to the isthmus. In both cases, electrodes with small RVI values correspond to the region of low RVIs obtained on the high-density grid in Fig. 2C (minimum interpolation with R = 4 mm - top right panel). In Fig. 8C, the decapolar catheter is arranged in a fan-like structure. Note that only one electrode is within the vulnerable region, but it is still capable of identifying the critical region. The lowest RVI in all three catheter arrangements become less negative (−108 ms on average) than in the high-density case (−128 ms). Fig. 8D shows RVI computed on clusters of recording sites randomly distributed across the tissue surface to reflect clinical data collection with a multipolar catheter.

RVI values are negative in almost all electrodes inside the critical region. Only one electrode placed on the lowermost part next to the stimulus site has a RVI larger than 50 ms. This is because this electrode has no downstream neighbor (within a 4 mm radius) located on the other side of line of block. Similar results were obtained when using the nearest neighbor and average interpolation to construct the RVI maps using the catheter arrangements in Fig. 2A–C (data not shown), but with less negative lowest RVI values. In the case of random grids, both the nearest neighbor and average interpolation were again more specific (smaller number of electrodes with negative RVIs) than the minimum.

**Fig. 9:**
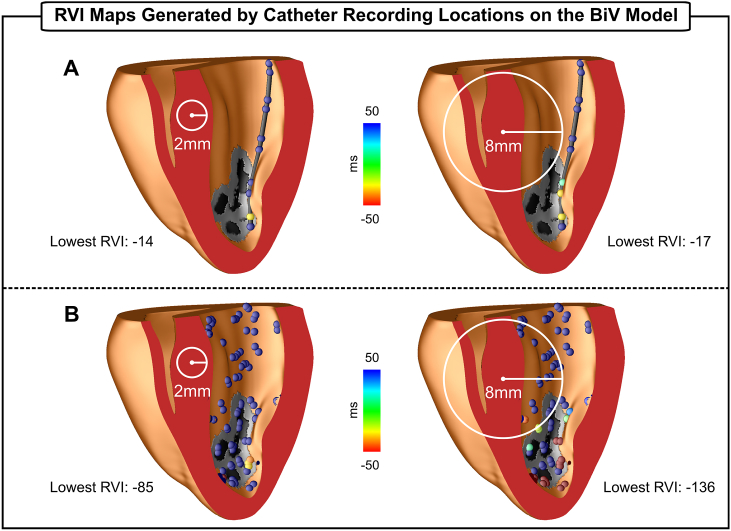
RVI maps computed on different electrode arrangements placed on the LV of the BiV scar anatomy model. A) Decapolar catheter inserted into the LV. B) Multipolar catheters placed randomly on the LV endocardium. Maps were constructed with R = 2 mm (left panels) and R = 8 mm (right panels). The minimum interpolation was used to construct the maps.

The feasibility and potential of RVI mapping in clinically-relevant conditions related to scar anatomy and irregular ventricular geometry was also investigated in the BiV scar anatomy model. Fig. 9A and B show RVI maps of the S2 beat computed on a decapolar catheter (electrode spacing rescaled to 1.25-5-1.25 mm to fit in the rabbit heart) inserted in the left ventricle and on multipolar catheters arranged randomly, respectively. It can be seen in Fig. 9A that only one electrode (in yellow) has a small RVI value as it is the only measurement point inside the critical area with a downstream neighbor within a search radius of 2 mm. Similar results were obtained using the nearest neighbor interpolation, whereas no negative RVI values were observed when using the average and R = 8 mm (data not shown). Although in the case of the multipolar catheter arrangements (Fig. 9B) there are more points within the critical area (see Fig. 6C - top/right panel), these do not have small RVI because: 1) there are not neighbors spanning the line of block within R; 2) electrodes are located on the scar which has no AT or RT as it is non-excitable tissue. Furthermore, in both scenarios, the smallest RVI -14 ms (decapolar catheter) and −85 (multipolar catheter) are more positive compared to −104 ms in the high-density grid (Fig. 6C - top/right panel). Increasing R from 2 mm to 8 mm increased the number of measurement points with low RVI values. Both nearest neighbor and average interpolation produced slightly more specific results when R = 2 mm is used (data not shown). The nearest neighbor when combined with a larger R (8 mm) led to a more blurred RVI map, similar to the 2D case in Fig. 5A (lowermost panel). All RVIs obtained with the average interpolation and R = 8 mm were positive (data not shown).

### 3.6 Assessing the ability of rapid steady-state pacing for use within the RVI protocol

**Fig. 10:**
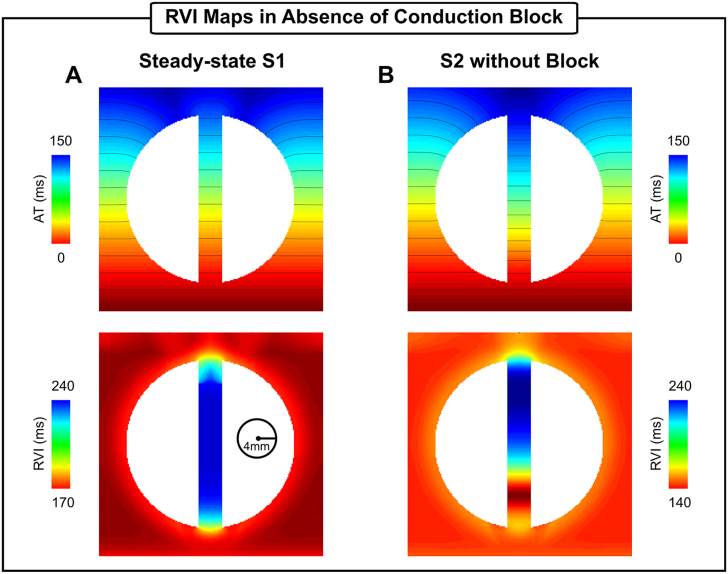
RVI maps constructed using different pacing protocols. A) ATs and RVI map in steady-state S1 pacing at a BCL of 500 ms. B) ATs and RVI map in a premature S2 beat (coupling interval of 250 ms) without conduction block. The minimum interpolation and R = 4 mm were used to compute RVIs.

**Fig. 11:**
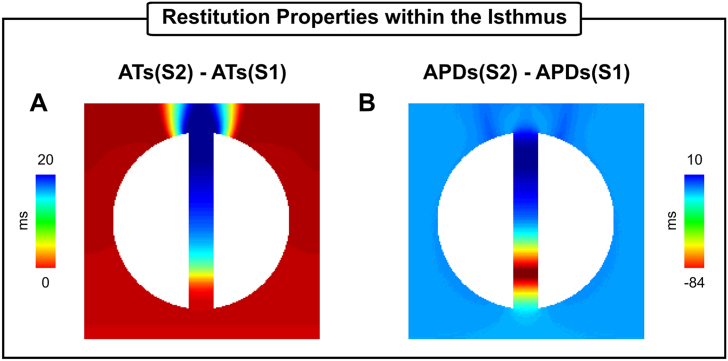
AT and APD restitution properties of the 2D idealized infarct model. A) Differences between ATs of the S2 and S1 beats. B) Differences between APDs of the S2 and S1 beats.

To investigate the behavior of the RVI method in absence of conduction block, simulations with non-arrhythmogenic (fast, but steady-state) pacing protocols were performed within the 2D idealized infarct model. Fig. 10 presents results obtained with steady-state S1 pacing (BCL = 500 ms) as well as with a premature S2 beat with a coupling interval of 250 ms. During S1 pacing the RVI maps resemble the APD profile of the tissue (as discussed in the Methods), which in this setup is longer inside the isthmus, 229 ms ± 9 ms, than in the myocardium: 176 ms ± 4 ms (see local AP traces during S1 pacing in Fig. 2C and the tissue RVI map in Fig. 10A). In the S2 without block (Fig. 10B) a region of smaller RVIs (140 ms) compared to the values computed in the myocardium (160 ms on average) can be seen at approximately 25% into the channel. This is because of restitution differences between the BZ and healthy myocytes as highlighted in Fig. 11. The premature S2 beat travels slower inside the isthmus (Fig. 11A) leading to later ATs, up to 20 ms, when compared to the S1. A relatively larger difference can be seen in the APD map shown in Fig. 11B. Cells in the BZ have a longer APD than the myocardium during steady-state pacing. However, the diastolic interval is shortened in these cells during the faster S2 beat which results in the APD shortening at the proximal mouth.

### 3.7 Electrophysiological properties of the infarct BZ

**Fig. 12:**
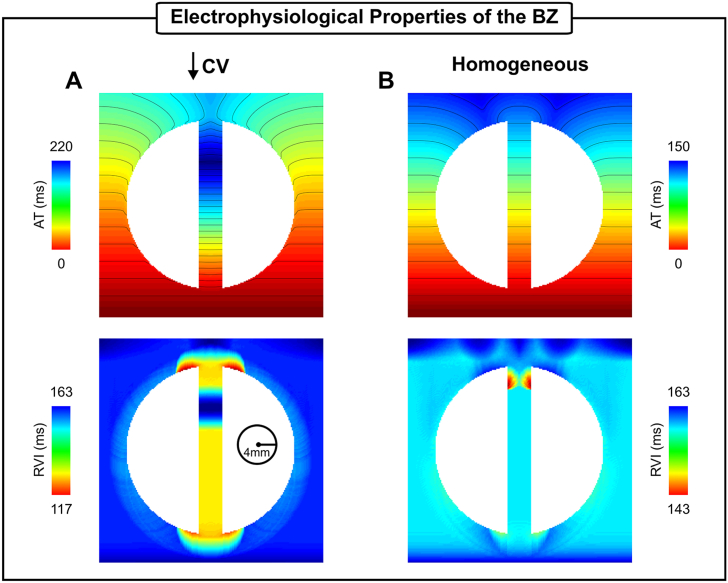
Effects of different electrophysiological properties of BZ tissue on the RVI metric. ATs and RVI maps following an S2 beat with a coupling interval of 250 ms in 2D infarct models with A) Slow conduction in the BZ; and B) Homogeneous tissue with same properties of the healthy myocardium.

Due to the heterogeneity in the electrophysiological remodelling of the BZ at different stages of infarct healing and across different species, the influence of different electrophysiological properties of the BZ on RVI maps were evaluated in this work. Fig. 12A shows simulated ATs and RVI maps in an idealized model, where a slower conduction velocity (↓ CV) was prescribed in the BZ. In addition, a homogeneous scenario where there are no differences between myocardium and BZ was included (Fig. 12B). The maps presented in Fig. 12 were computed following an S2 beat in absence of conduction block (coupling interval of 250 ms). RVI values within the isthmus in the ↓ CV scenario (139 ms ± 9 ms) are smaller than the homogeneous case (155 ms ± 2 ms) as well as the long APD setup (203 ms ± 34 ms) in Fig. 10B. This is because the difference between RTs and ATs in the presence of slow conduction is smaller (AT is increased). Note that in the upper part of the isthmus the RVI is similar to the myocardium despite the reduced CV. This is caused by a collision of slow propagating wavefronts from both mouths of the canal. In the homogeneous case shown in Fig. 12B, gradients in the RVI map can be seen near both mouths. The RVI is smaller at the distal mouth due to electrotonic effects that slow down conduction velocity at regions of tissue expansion.

## 4 Discussion

In this study we used computational models to thoroughly assess the ability of the RVI algorithm to identify regions vulnerable to VT under clinically-relevant mapping conditions. Simulations showed that the specificity and sensitivity of the RVI maps depend more on the size of R than on the type of interpolation chosen. For small radii the interpolation does not affect the ability of the RVI to detect vulnerable regions. However, large R might result in under- (average interpolation) or overestimation of ablation targets (minimum interpolation). Noise had a minor effect on RVI maps, in particular when using the minimum interpolation. Furthermore, our results demonstrated that the RVI performs well even when constructed on sparse recording grids. Accurate identification of the vulnerable region was possible with only two recording electrodes: a single one located inside the region and second downstream neighbor. Finally, in fast pacing without conduction block the RVI was not capable of precisely determine VT isthmuses, but may provide valuable information on altered repolarization and/or slow conduction.

### 4.1 Sensitivity and specificity of RVI maps to detect vulnerable regions

Precise identification of targets for catheter ablation of VTs is still a clinical challenge [3,4]. Re-entrant circuits sustaining VTs involve a complex interaction between the tail (repolarization) of the wavefront and the head (activation) of the following wavefront. A re-entry can only be formed if the wavelength, the mathematical product of the conduction velocity and the effective refractory period (largely determined by APD), is shorter than the length of the re-entrant circuit [28]. The portion of tissue capable of being re-excited or the “excitable gap” allows a re-entrant wavefront to continuously propagate around the circuit. Its size is affected by conditions promoting slow conduction or APD shortening [28]. The longer the excitable gap, the more likely it is for a premature stimulus to initiate a re-entry.

As the RVI measures the difference between RT and AT of two points spanning the line of block, it defines whether a wavefront that attempts to re-enter on its first cycle will be able to successfully do so. A positive RVI means that the wavefront collides with the tail and re-entry is not possible (tissue at the site of initial block remains refractory), whereas a negative RVI means that the tissue at the site of initial block has recovered prior to the arrival of the wavefront, and re-entry can occur. Thus, the RVI can be thought of as quantifying the temporal size of the excitable gap on the first attempted cycle of re-entry. Should CV be known/measured, this could also be translated into the existence of a physical excitable gap length.

However, differences in the implementation of the RVI algorithm might lead to under or overestimation of the vulnerable regions. Specifically, while the definition of the RVI metric between a point at the recording site and a single one of its downstream neighbors is well defined [17], how RVIs associated with the same recording site are interpolated to create a map was not investigated. Successive values of the RVI from successive pairs (corresponding to different downstream neighbors) combined to give a single RVI value associated with the recording site in question may significantly change the quantitative features of the RVI map, and, more importantly, its ability to successfully highlight regions of re-entry susceptibility. Indeed, differences in the algorithm parameters altered the specificity and sensitivity of the RVI maps as shown in Fig. 5, Fig. 6 and Table 1, Table 2, Table 3, Table 4, where maps were constructed using the nearest neighbor [15,19], average and minimum [18]. On one hand, RVI maps computed on high-density grids (each finite element node in the meshes in this study) with small R could identify the VT isthmus with high specificity regardless of the interpolation used (see size of the vulnerable region in Table 1 and Table 3). Ablation of these specific locations have been demonstrated to successfully terminate re-entry [19]. On the other hand, if a large R (≥ 16 mm) is used as in clinical studies [18], areas of small negative RVIs might be missed (average interpolation in Figs. 5B and 6B) or overestimated (minimum interpolation in Figs. 5C and 6C). Since radiofrequency ablation lesion size ranges from 5.7 mm to 9.4 mm in diameter [29], overestimated targets could lead to large lesions that can further impair ventricular function.

### 4.2 RVI algorithm to predict ablation targets in simulation-guided strategies

Recently, simulation-based strategies have been employed to predict risk of sudden cardiac death as well as to identify optimal targets for anti-arrhythmia ablation therapies [20,30]. In retrospective and prospective studies, Prakosa et al. [30] demonstrated the capability of computational modelling to improve scar-related VT ablation guidance. RVI maps constructed on high resolution heart models such as the BiV in Fig. 6 could be readily combined with such non-invasive *in-silico* approaches for patient-specific treatment planning. Simulation-based approaches could help reducing the clinical procedure time since the pacing/mapping protocol will not be necessary during the therapy. Moreover, complex scar anatomies such as those in the BiV model (Fig. 3A) may support different re-entrant circuits that should be targeted to prevent later recurrence of VT. Pacing the heart from multiple locations can reveal other pathways through the scar and has been shown to change RVI hot-spots [19]. Unlike during the clinical procedure where the number of pacing locations is limited, the virtual heart can be paced from multiple sites. RVI maps constructed from multi-site pacing on personalized models could be imported into the clinical electro-anatomical navigation system to guide clinicians towards the ablation targets in a comprehensive way.

### 4.3 RVI maps constructed on noisy AT and RT data

In this work ATs and RTs were obtained from AP as illustrated in Fig. 4A. In the clinical setting, these markers are commonly obtained with electrophysiology catheters that record unipolar electrograms that can be fractionated or contaminated by noise. In the presence of noise, the calculation of AT and RT should be performed with appropriate filtering techniques since they can affect the RVI map. Even in a worst-case scenario represented in Fig. 7 where noisy AT and RT data were not filtered, the vulnerable regions could still be localized in all RVI maps. The RVI map constructed with the minimum interpolation in particular was the less affected by noise suggesting that this approach might be preferred in the clinic.

### 4.4 RVI in clinically-relevant mapping conditions

Accurate location of VT circuits during catheter ablation procedures depends on signal sampling that is typically done with manual manipulation of recording catheters. Advances in electro-anatomic mapping systems allow the collection of multiple signals at the same time, but grid density (distance between electrodes) remains low (∼ 2.5 mm) [31]. The ability of RVI maps constructed based on RT and AT data from regular low resolution measurement grids has been investigated previously using the nearest neighbor interpolation [19]. Hill et al. [19] showed that, for a fixed R, overall spatial region of reduced RVI values remains unchanged even at very low resolution (8 mm electrode distance). However, clinical recording grids are not regular but sparse and randomly distributed as data collection depends on manual placing of multipolar catheters as illustrated in Fig. 8, Fig. 9. Moreover, in this scenario, where the number of measurement points is reduced, the minimum interpolation might be preferred due it its higher sensitivity [18] (see Figs. 5C and 6C). RVI maps constructed on 2D sparse grids resembling different placements of a recording catheter (see Fig. 8) can still produce a qualitatively similar RVI map to that of a high density grid (Fig. 5). This is also the case for RVI maps computed on electrodes placed on the sparse and irregular endocardial surface of the BiV model in Fig. 9. The results in Figs. 8C and 9 demonstrate that only one electrode inside the vulnerable region is still capable of identifying the critical region as long as it has a downstream neighbor within R. Furthermore, Hill et al. [19] also showed that the lowest RVI value becomes less negative and even becomes positive as resolution is increased. This is in agreement with our findings where the lowest RVI increased from −104 ms (Fig. 6C, R = 2 mm) to −14 ms (Fig. 9A, R = 2 mm). Similar results were also obtained with the nearest neighbor and average interpolation (data not shown). Finally, the size of R will depend on the density of the recording grid. The analysis presented here suggests that small values of R (≤ 4 mm) might be preferred in simulation-guided ablation [30] due to the high density of the measurement grid, whereas larger R (> 4 mm) may be more suitable for clinical procedures [15,18].

### 4.5 Prediction of vulnerable sites without conduction block

Identification of re-entrant circuits commonly rely on VT induction [4]. However, mapping is limited in patients where inducibility is not possible or with haemodynamically intolerable VT. In these scenarios, substrate-base mapping strategies during sinus rhythm or pacing have been used as alternatives to the conventional arrhythmia mapping [3,4,31]. Characterization of the abnormal substrate relies on the identification of areas of slow conduction or with altered electrogram characteristics [[5], [6], [7]] suggesting abnormal conduction [[8], [9], [10], [11]]. The potential of the RVI as a substrate mapping procedure has been investigated in Fig. 10, Fig. 11, Fig. 12. During steady-state pacing (sinus rhythm), RVI values reflected the APD heterogeneity in tissue (Fig. 10A) rather than highlighting the region of conduction block resulting from a fast S2 beat (Fig. 5). Although regions of small RVI values alone should not be used as ablation targets in this scenario, the RVI map may provide additional information about dispersion of repolarization that is known to be associated with a higher risk of VT [16,32,33]. In the 2D infarct model shown in Fig. 10A this would correspond to both mouths.

In addition to mapping during sinus rhythm, AT and RT collected following fast ventricular pacing can uncover restitution differences in cardiac tissue that can lead to conduction block. Although block and re-entry were not induced by the S2 beat in Fig. 10B, RVI values about 15% smaller (140 ms) than the average RVI (164 ms) in the myocardium were observed at approximately 25% into the isthmus mouth proximal to the stimulus site. Differences in AT and APD restitution properties, as shown in Fig. 11B, may explain regions of small RVIs in absence of VT. In the idealized infarct model, the shorter diastolic interval at the proximal mouth compared to the rest of the isthmus resulted in a shorter APD (−84 ms when compared to the S1 beat). This finding suggests that high-resolution RVI maps can help to identify entry/exit sites during rapid pacing in cases where S1-S2 pacing with block is not possible. In the clinical setting, on the other hand, small RVIs could arise due to difficulties in processing data from coarse recording grids and noisy/fractionated signals. However, these differences have become smaller and smaller as the mapping resolution of catheters continues to increase.

Albeit the RVI is defined as an absolute time difference, its interpretation will depend on whether conduction block occurred and whether the vulnerable region was minimally mapped by the recording system. This is apparent when considering the different ranges spanned by the color bars of the RVI maps in Fig. 5, Fig. 6, Fig. 7, Fig. 8, Fig. 9, Figs. 10 and 12. Absolute RVI values depend on the presence of VT (negative RVI as in Fig. 5, Fig. 6, Fig. 7, Fig. 8, Fig. 9), block without re-entry (small but positive [15,18]), or be similar to the tissue APD in case of normal conduction (Fig. 10). Furthermore, activation-repolarization dynamics differ from patient to patient and have also been shown to have a strong dependency on the pacing cycle length [34], which will also influence the specific value of the RVI for a given scenario. In a recent clinical study, Martin et al. [18] have addressed these issues by looking for areas with relative lower RVI values than the rest of the heart. The authors have sought for the bottom 5% of all RVI values as a relative reference to identify critical regions. They showed that such regions co-localized with the VT earliest activation site in patients with right ventricular pathology such as Brugada Syndrome and Arrhythmogenic Right Ventricular Cardiomyopathy [18]. Although, this and other clinical work have shown that a train of S1 pulses followed by a S2 consistently provided some degree of block without inducing VT [15,18], the proposed relative threshold might lead to the detection of “false positives”, particularly in absence of conduction block (see Fig. 10). Therefore, a combination of a relative and an absolute threshold based on the tissue APD may be desired to avoid misdetection of healthy myocardium as a vulnerable region.

### 4.6 Effect of different remodelling stages of the BZ on RVI maps

The computational models used here follow recent simulation studies with image-based models of the infarcted heart, where cells in the BZ are modelled with longer APD [20,30]. However, the electrophysiological properties of the BZ vary significantly depending on stage of the infarct healing (see Costa et al. [23] for a thorough review). The influence of ionic remodelling (represented here by APD prolongation) and structural remodelling (slow conduction) of the BZ on RVI maps were evaluated in this study.

RVI values were shown to be larger within an isthmus with abnormally long APD (Fig. 10B) and shorter in the slow conducting isthmus (Fig. 12A). The results in Fig. 12A suggest a possible correlation between reduced CV and low RVI. However, in the upper part of the isthmus the RVI is similar to the rest of the myocardial. This region of “normal” RVI results from the collision between the slow wavefront traveling from the proximal mouth with a second one entering the isthmus from its distal mouth. In absence of remodelling (Fig. 12B), the RVI map resembled small dispersions in APD generated solely by structural effects [35]. The lowest RVIs were found at the distal mouths of both ↓ CV (Fig. 12A) and homogeneous isthmuses (Fig. 12B). This is caused by electrotonic modulation that slows conduction at regions of rapid tissue expansion. Interestingly, Anter et al. [31] showed that the VT critical zone corresponds to regions with steep activation gradients during sinus rhythm.

Unlike the RVI maps in Fig. 5, Fig. 6, Fig. 7, Fig. 8, Fig. 9, where VT was induced by S2 beat with a shorter coupling interval, the lowest RVIs in Figs. 10 and 12 are positive and higher. Nonetheless, the results demonstrated that the RVI in absence of VT can still provide important information about the electrophysiological remodelling in sites thought to be part of the VT circuit.

### 4.7 Limitations

Although the computational models used in this study are state-of-the-art, they are a simplified representation of the structural heterogeneity of the infarct BZ [36]. Only one idealized infarct model with a fixed isthmus’ width was presented in the investigations here. Additional simulations in idealized models with varying isthmus widths were performed (data not shown) to investigate a possible association between the width of the isthmus and the values of R. However, no apparent relation between these two parameters were observed. Similarly, the feasibility and potential of RVI metric in more clinically-relevant scar anatomies was only studied in one realistic BiV model. The aim of this work was to compare RVI maps computed on high-resolution grids to maps computed on more realistic mapping conditions to further develop the RVI for its use in a clinical protocol. Therefore, including additional high-resolution RVI maps from other scar anatomy models and compare them to their respective low-density ones would not change our main findings. The use of idealized and BiV scar anatomy models allowed a detailed analysis of the RVI metric under clinically-relevant conditions while avoiding the computational burden associated with highly detailed ventricular models. Nevertheless, all computational analysis here could be applied to patient-specific clinical models to investigate any potential differences. Furthermore, following previous simulation studies [19,20] ionic properties of cells within the BZ were adjusted to prolong APD. However, experimental data show that the electrophysiological properties of the BZ at the chronic stage are dominated by structural remodelling mostly marked by the presence of fibrosis and fiber disarray [23]. The focus of the present study was to assess whether the RVI metric could identify the vulnerable regions under the recording conditions mimicking the clinical scenario. A detailed investigation into the mechanisms of scar-related VTs is out of the scope of this work. While the region vulnerable to block and re-entry, or the “ground truth” in this study, could be readily detected by visual inspection of the premature S2 wavefront in the 2D idealized model, this is not trivial in more realistic setups such as the BiV scar anatomy model. Nonetheless, the ability to successfully terminate VT by ablating regions of low RVI values has been demonstrated in previous computational and experimental studies. The efficacy of RVI to accurately identify vulnerable regions compared to other mapping methods in patient-specific models is an avenue to be pursued in the future work.

## 5 Conclusions

The RVI has been shown to be able to reliably predict sites vulnerable to VT in a series of experiments. Here we employed computational models to optimize the RVI algorithm under clinically-relevant mapping conditions. Within an idealized 2D infarct model as well as in a realistic BiV model we showed that for small search radii, all interpolation methods can identify with high specificity and varying sensitivity the isthmus maintaining the VT. This finding suggests that the RVI could potentially further improve the prediction of optimal ablation targets of simulation-based strategies. Our results also showed that the minimum interpolation could result in larger ablation lesions if combined with a large search radius. RVI maps computed on sparse and irregular recording grids also identified vulnerable regions as long as two electrodes were placed on both sides of the line of block. Furthermore, although RVI maps generated in absence of VT could not pinpoint the location of re-entrant circuits, they provided additional information about dispersion of repolarization and slow conduction that could be valuable during catheter ablation.
